# The Influence of Doctors’ Online Reputation on the Sharing of Outpatient Experiences: Empirical Study

**DOI:** 10.2196/16691

**Published:** 2020-12-11

**Authors:** Yang Wang, Hong Wu, Xueqin Lei, Jingxuan Shen, Zhanchun Feng

**Affiliations:** 1 School of Medicine and Health Management Tongji Medical College Huazhong University of Science and Technology Wuhan China

**Keywords:** online health communities, individual reputation, doctor reputation, patient feedback, organizational reputation, disease severity

## Abstract

**Background:**

The internet enables consumers to evaluate products before purchase based on feedback submitted by like-minded individuals. Displaying reviews allows customers to assess comparable experiences and encourages trust, increased sales, and brand positivity. Customers use reviews to inform decision making, whereas organizations use reviews to predict future sales. Prior studies have focused on manufactured products, with little attention being paid to health care services. In particular, whether patients prefer to use websites to discuss doctors’ reputation has so far remained unanswered.

**Objective:**

This study aims to investigate how patient propensity to post treatment experiences changes based on doctors’ online reputation (medical quality and service attitude) in delivering outpatient care services. Further, this study examines the moderating effects of hospitals’ (organizational) online reputation and disease severity.

**Methods:**

Fractional logistic regression was conducted on data collected from 7183 active doctors in a Chinese online health community to obtain empirical results.

**Results:**

Our findings show that patients prefer to share treatment experiences for doctors who have a higher medical quality and service attitude (β_service attitude_=.233; *P*<.001 and β_medical quality_=.052; *P*<.001) and who work in hospitals with a higher online reputation (β=.001; *P*<.001). Patients are more likely to share experiences of doctors who treat less severe diseases, as opposed to those treating severe diseases (β=−.004; *P*=.009). In addition, hospitals’ online reputation positively (negatively) moderates the relationship between medical quality (service attitude) and patient propensity to post treatment experiences, whereas the moderating effects of disease severity on doctors’ online reputation are negative.

**Conclusions:**

Our research contributes to both theory and practice by extending the current understanding of the impact of individual reputation on consumer behavior. We investigate the moderating effects of organizational reputation and consumer characteristics in online health communities.

## Introduction

### Background

In seeking health care provision, patients often face uncertainty regarding the quality of doctors’ services, lacking trustworthy channels for accessing information such as medical quality and bedside manner [1]. Medical quality has historically been associated with treatments received by hospitals at the organizational level; however, patients are increasingly seeking information relating to the quality of individual doctors, that is, at the doctor level. Information asymmetries between patients and doctors exist extensively, with patients now regularly interacting on social networking sites to inform their provision needs based on peer recommendations. With the growing popularity of web 2.0 technologies, online health care communities provide a useful channel for patients to obtain doctors’ information. In China, more than 80% of patients search for health care information before visiting hospitals [2].

Internet-based media play an important role in providing prepurchase information and informing decisions. These burgeoning new media have been hailed as a democratizing force that enables consumers to discuss products and services online [3]. In online communities, consumers critically evaluate the quality of comparable products by analyzing brands, pricing, and retailer reputation (note: in this paper, we use treatment experiences, reviews, word-of-mouth, and feedback interchangeably, as well as patients, consumers, and service receivers), which act as signals of product quality [4,5]. Prior literature suggests that a higher reputation can also signal higher quality [6,7]. Numerous empirical studies have suggested that reputation is one of the predominant factors influencing consumer purchases and seller performance [8,9], consistently revealing that there is a close link between consumer reviews and future sales. Sellers’ online reviews positively impact product demand [10], with reviews creating a bridge of communication between consumers and sellers, decreasing consumers’ perceived risk, and boosting trust and cooperation on both sides [11].

Reviews, which are generally agreed to be more effective than traditional advertising [12], increasingly affect consumer behaviors [13,14]. They facilitate the prediction of future performance and, therefore, are required more than ever by sellers. Existing literature shows that consumers who are pleased or displeased with a product will make their opinions known to others [15]; the more satisfied or dissatisfied the consumer, the more likely they are to post feedback about their experiences [16]. However, much less is known in relation to health care products and services. To the best of our knowledge, only Wu and Lu [17] have studied the role of doctors’ reputation and its influence on patient propensity to share reviews, identifying that doctors with a higher reputation receive a greater number of reviews. However, their study focused solely on the role of the individual doctor’s reputation and failed to consider the organizational reputation and consumer characteristics, which are important factors that affect consumer behaviors. According to the theory of psychological choice [18], the effect of a signal (individual reputation) is moderated by environmental situations (organizational reputation) and contextual factors (such as consumer characteristics), with final responses dependent on their interaction effects. This study aims to fill this critical gap by studying the impact factors of patient propensity to post treatment experiences online. We seek to understand and address the following questions:

- *Question 1*: How does a doctor’s reputation influence patient propensity to post treatment experiences online?
- *Question 2*: How does a hospital’s reputation moderate the relationship between the doctor’s reputation and patient propensity to post treatment experiences online?
- *Question 3*: How does disease severity moderate the relationship between the doctor’s reputation and patient propensity to post treatment experiences online?

We argue that reputation, signaled by existing reviews, can predict future reviews. Data were collected from an online health community, which, in recent years, has helped patients find doctors, book outpatient care services, and search for medical information. Unlike extant literature on manufactured products, our study includes both medical quality and service attitude, which are important factors in the health care field, as part of the doctor’s reputation in our model [19]. In recent years, patients have complained about doctors’ bad attitudes. Thus, in addition to improving service quality, it is vital to mitigate conflicts between doctors and patients by enhancing service attitude [20]. Moreover, we examined the moderating effects of hospital (organizational level) reputation and disease severity (patient characteristics). patient propensity to post treatment experiences is the ratio of the increment of the treatment experience to the increment of outpatient care service demands over a time window of interest.

### Online Health Communities

In recent years, online health care communities have been developed by patient organizations, medical service providers, and nonprofit organizations to make it easier for patients to find health-related information [21]. Such communities provide virtual forums for patients to obtain services and discuss treatment experiences. Researchers have started to investigate the benefits and user behaviors of such communities, from the perspective of doctors [19,22] and patients [23-25]. For doctors, Ni and Sun [26] studied the willingness of doctors to work on online platforms and associated benefits. For patients, Xiao et al [27] explored whether patients’ information search behavior influenced their perceived health condition. With regard to the impact factors of whether or not to post health information online, people take privacy and information sensitivity into consideration [28].

In China, as a result of continued limitations in existing health care provisions, online health communities have been strongly adopted by citizens. China has the world’s largest population and thus represents a huge resource-consumption country. China’s large population generates a variety of unique health care needs and, therefore, exhibits unique behaviors within online health care communities. Health ultimately concerns everyone, and with the emergence of online health care communities, patients now have more channels to find doctor information, whereas doctors have more choices in the way they deliver medical treatment. On the basis of extant literature, we have found few studies that explore the effects of doctors’ reputation on patient propensity to post treatment experiences and the moderating effects of hospitals’ reputation and disease severity. Our research, therefore, aims to fill these gaps.

### Theory of Psychological Choice

Hansen [18] presented an overview of psychologists’ approaches to consumer choice and the processes employed in different scenarios. He determined that the consumer choice process is characterized by conflict, uncertainty, cognitive activity, and related psychological processes. Individuals’ choices depend on their current situation, whereas the nature of the problem can be described as a comprehensive result of internal and external factors. Behavioral response is a result of the interaction between environmental factors and individual characteristics such as values, beliefs, intentions, and preferences. According to the theory of psychological choice [18], consumer behavior, dependent on circumstances, is decided by a series of factors, including internal and external factors. The effect of a signal is moderated by environmental situations and contextual factors, such as consumer characteristics, with final responses being decided by the interaction effects of these factors.

### Expectation-Confirmation Theory

Expectation-confirmation theory is widely used to explore consumer behavior in both product marketing [29] and service marketing [30]. Oliver [31] described in detail the process of expectation, confirmation, and postpurchase behaviors. First, consumers form an initial expectation of the product or service, which is shaped by personal experiences, norms, and the present environment [32]. Second, consumers form perceptions about performance after receiving the product. Third, consumers will assess their pre-expectation and perceived performance and estimate the gap (degree of confirmation) between expectation and perceived performance. Fourth, the confirmation between pre-expectation and perceived performance influences their satisfaction and ultimately determines their future behaviors, such as repurchase intention and word-of-mouth.

### Research Hypotheses

**Figure 1:**
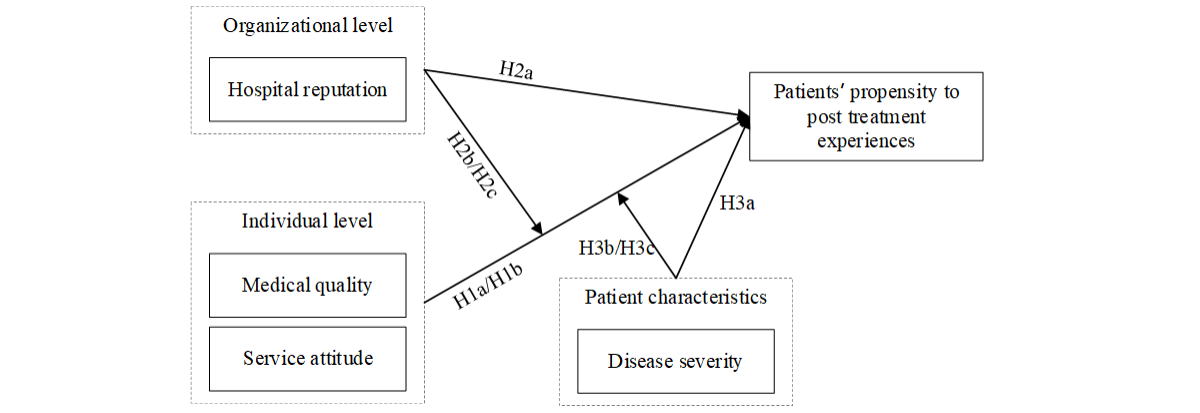
Conceptual model.

Consumer reviews are an important criterion that impacts consumer behavior. However, existing literature rarely investigates the relationship between doctors’ online reputation and patient propensity to post treatment experiences online. We sought to examine how doctors’ medical quality and service attitude affect their patient propensity to post treatment experiences. Moreover, we attempted to investigate the moderating effects of the hospital’s reputation and disease severity. Figure 1 shows the conceptual model of this study.

Nowadays, the internet enables consumers to easily post opinions and express thoughts, feelings, and viewpoints on products and services to the wider online community [33]. On the basis of the expectation-confirmation theory, high reputation enhances consumers’ expectations of quality and vice versa [34]. Pre-expectation will be compared with the perceived and/or actual performance received following the purchase of a product or service. Compared with low expectations, a high expectation is less likely to be reached by perceived performance [35]. The degree of confirmation between expectation and perceived performance dictates consumer satisfaction [31]. For consumers, the primary motivation to share positive or negative comments is to inform others [15] and/or to express their satisfaction or dissatisfaction [36]. Consumers are likely to express their feelings to others when their expectations are either surpassed or not met [37], and the propensity to post online reviews is greater for extreme experiences but smaller for average experiences [38].

In online health communities, patients hold comparatively high expectations about service quality for doctors with a high reputation. High expectations are less likely to be reached by perceived quality. The degree of expectation would affect consumer satisfaction and their propensity to post about treatment experiences. Higher expectations cause patients to be easily disappointed and dissatisfied after receiving services, which leads to them sharing negative feelings with others online [15,34]. The present literature has indicated that the existing reputation has a positive impact on the number of future reviews received [39-41]. However, the potential mechanism is unexamined in online health communities. On the basis of the abovementioned insights, we hypothesize the following:

- *Hypothesis 1a*: Doctors’ medical quality positively affects patient propensity to post treatment experiences online.
- *Hypothesis 1b*: Doctors’ service attitude positively affects patient propensity to post treatment experiences online.

An organization’s reputation helps consumers make informed choices when they feel uncertain about a product or service [42]. Organizational reputation strongly influences consumer expectation and purchase intention [43]. In product marketing, Amblee and Bui [39] demonstrated that the amount of future online reviews has a positive correlation with a product’s existing brand (organizational) reputation. In online health communities, a hospital’s reputation can be considered a signal to patients. Patients would have higher expectations from doctors who work in hospitals with high online reputations. Higher expectations more easily induce disconfirmation between pre-expectation and perceived performance, which would enhance patient propensity to post treatment experiences.

- *Hypothesis 2a*: A hospital’s reputation positively affects patient propensity to post treatment experiences online.

On the basis of the theory of psychological choice [18], the effect of a signal is influenced by environmental situations. As the medium for diffusing the signal, environments can influence the strength and effectiveness of the signal [44]. Many studies have examined the effect of signals in different environments and obtained consistent conclusions that the strength of a signal is moderated by signal environment uncertainty [45]. Therefore, the impact of individual reputation can be moderated by organizational reputation. A positive evaluation of an organization’s reputation generates a positive evaluation of an individual’s reputation [46].

With regard to online health communities, a hospital’s reputation can be treated as an environmental factor. The delivery process of a signal varies among different hospital environments. Thus, the hospital’s reputation can moderate the effect of a doctor’s reputation. In reducing patients’ perceived risks and increasing trust in the doctor’s reputation, a higher hospital reputation can make patients have a higher expectation about doctors’ performance. On the basis of the expectation-confirmation theory [34], higher expectation is less likely to be reached by perceived performance [35]. Patients will be more disappointed after receiving the service and are more likely to express their feelings to others online [15,34]. On the basis of the abovementioned insights, we develop the following hypotheses:

- *Hypothesis 2b*: A hospital’s online reputation positively moderates the relationship between a doctor’s medical quality and patient propensity to post treatment experiences online.
- *Hypothesis 2c*: A hospital’s online reputation positively moderates the relationship between a doctor’s service attitude and patient propensity to post treatment experiences online.

On the basis of the theory of psychological choice [18], the effect of a signal is also influenced by contextual factors. The influence of reputation varies with different types of products and services [47]. Individual characteristics significantly affect the degree of satisfaction with service quality [48] and moderate the relationship between service quality and satisfaction [49].

In the health care field, patient behavior is also influenced by their characteristics. Disease severity is an important basis for distinguishing between patients. Prior research has indicated that disease severity moderates the doctor’s reputation on the patient’s purchasing behavior [19]. It is argued that patients with severe diseases prefer to choose doctors with high medical quality rather than service attitude [19]. The study mainly focuses on the consumer buying process, but the decision on posting feedback is neglected.

From a positive perspective, disease severity may influence the patient’s physical and mental health [50]. Patients with severe diseases are more sensitive to the doctor’s reputation than those with less severe diseases. For example, compared with patients with less severe diseases, such as the common cold, patients with tumors are likelier to choose a doctor with a higher reputation and form higher expectations regarding their desire for health. Moreover, patients with severe diseases experience more pain and distress and are eager to find higher quality services.

From a negative perspective, patients with severe diseases often concentrate less on service attitude [51]. They prefer to choose doctors with higher medical quality rather than service attitude [19]. Purchasing preference leads to different feedback behaviors. Service attitude has fewer effects on patient propensity to post treatment experiences for patients with severe diseases. However, as a highly professional service, medical service sets an invisible barrier for patients who generally lack professional medical knowledge to assess medical quality, especially for severe diseases. Patients with severe diseases may not evaluate perceived quality objectively, thereby leading to a lack of certainty of disconfirmation between pre-expectation and perceived quality. In fact, given the issues they focus on, patients with severe diseases are more likely to have concerns about their recovery time and health, rather than posting feedback or complaining about poor experiences online. Furthermore, privacy concerns and information sensitivity, 2 critical influencing factors for deciding whether or not to spread health information online [28], are concerned by patients with severe diseases, ultimately decreasing patient propensity to post treatment experiences online.

On the basis of the aforementioned insights, we plan to determine the advantages of these effects in specific contexts. We propose both positive and negative moderating effects of disease severity:

- *Hypothesis 3a*: Disease severity significantly affects patient propensity to post treatment experiences online.
- *Hypothesis 3b*: Disease severity significantly moderates the relationship between a doctor’s medical quality and patient propensity to post treatment experiences online.
- *Hypothesis 3c*: Disease severity significantly moderates the relationship between a doctor’s service attitude and patient propensity to post treatment experiences online.

## Methods

In this section, we describe the research context and data collection process and present the variables and models.

### Research Context

We test our hypotheses using data collected from the WeDoctor website, a leading online health community authorized by the Chinese Health and Family Planning Committee. WeDoctor has become the leading online health community in China, mainly providing appointment booking services for outpatient care. The website helps increase efficiency for both patients and hospitals. Using the WeDoctor website, patients can easily make appointments and save valuable time. By 2020, the community has helped more than 850 million citizens. The WeDoctor website started to provide online written consultation and video consultation services in September 2016. In our proposed model, we do not include written and video consultation services for 2 reasons. First, compared with the outpatient care service appointment function, written and video consultation services are rarely used by patients. Second, our data were collected in the first half of 2016 when only outpatient care appointment services were provided by the website.

More than 7800 hospitals and 260,000 doctors are active in the online community. WeDoctor creates home pages for doctors and their hospitals. Doctors can self-manage their home pages, including modifying schedules for outpatient care services and updating individual information. The website has a formal and comprehensive reputation mechanism, which is important for this study. Patients can post their treatment experiences after receiving outpatient care services in the hospitals. Treatment experiences help future patients make better choices.

### Sample and Data Collection

We used a crawler to automatically download doctors’ information from the WeDoctor website using the following selection criteria. First, we crawled all active doctors who usually add or modify their outpatient care service information or other individual information (active doctors are recognized by WeDoctor). Second, we selected doctors who treat severe diseases and who treat relatively less severe diseases. Severe diseases include malignant tumors and heart and cerebrovascular diseases. Less severe diseases include endocrine, digestive, and nervous system diseases. The reasons for choosing these disease categories will be explained in detail in the following section. We repeated the collection process in 2 time periods: one week in March 2016 and another week in June 2016. We included in our analyses the doctors who were seen at both collection times, yielding a sample of 7183 doctors. For each doctor, we collected their reviews, reputation, and other relevant information (eg, hospital information). We also collected information on the medical departments with which the doctors were affiliated.

**Figure 2:**
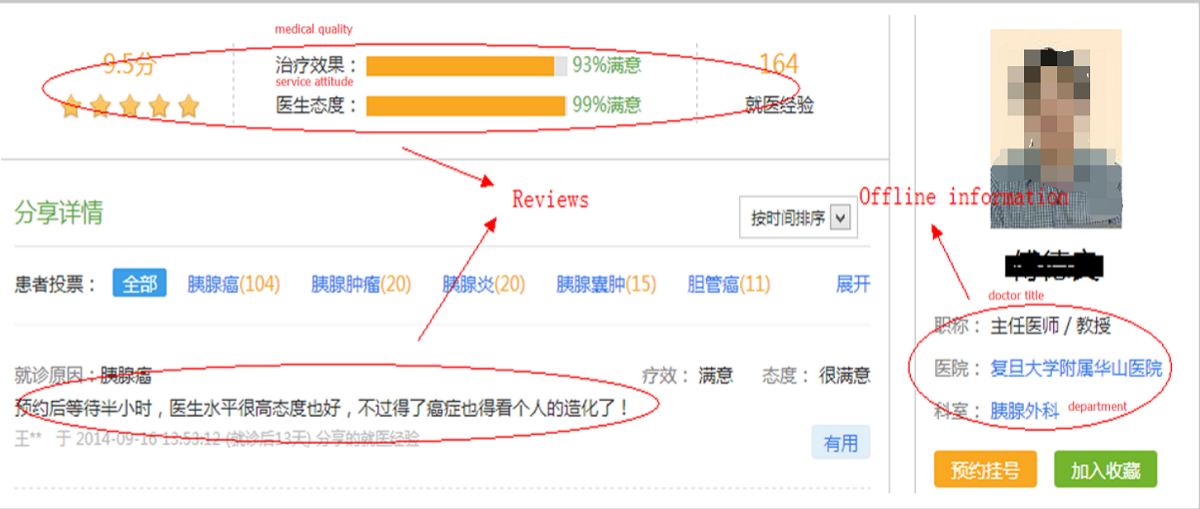
A doctor’s home page on the WeDoctor website.

**Figure 3:**
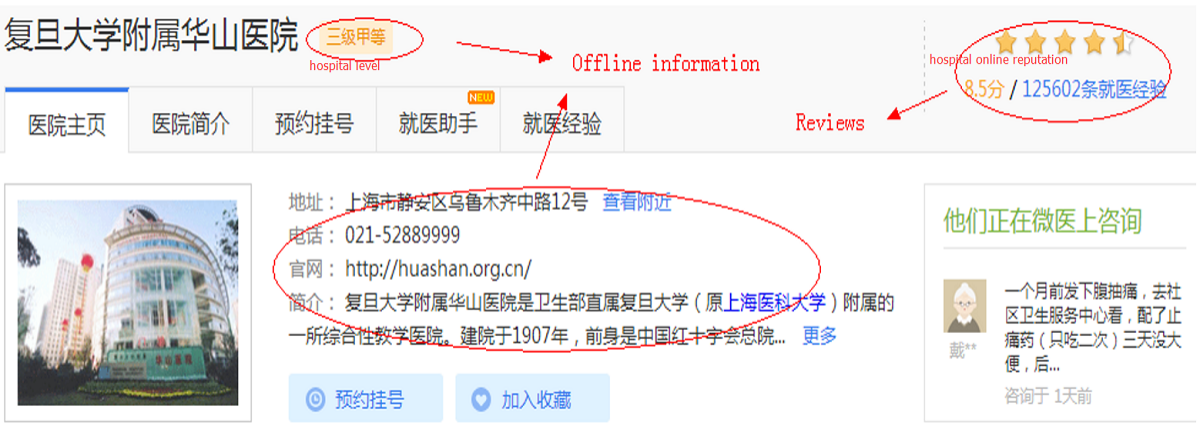
A hospital’s home page on the WeDoctor website.

From each doctor’s home page, we collected information posted about patients’ experiences. Each patient can give a score to the doctor’s medical quality and service attitude observed during treatment. Other patients can then read these reviews to make informed decisions. Figure 2 shows an example of a doctor’s home page, whereas Figure 3 shows an example of a hospital’s home page.

### Variables and Empirical Models

The variables used in this study are in the form of aggregated data at the doctor level, which can help control for the potential influence of patient heterogeneity [52]. The detailed definitions for all variables included in this study are shown in Table 1.

**Table 1:** Variable definitions.

Variable		Definition
**Dependent variables**		
	Patient propensity to post treatment experiences	The ratio of the increment of the treatment experience to the increment of outpatient care service demands over 3 months for each doctor.
**Independent variables**		
	Medical quality	Patients give an evaluation score for doctors’ medical quality when patients share treatment experiences. The WeDoctor calculates the mean of medical quality for each doctor based on all the existing treatment experiences posted by patients. The range of values for medical quality is from 0 to 1, with a greater value indicating a higher medical quality.
	Service attitude	Patients give an evaluation score for doctors’ service attitude when patients share treatment experiences. The WeDoctor calculates the mean of service attitude for each doctor based on all the existing treatment experiences posted by patients. The range of values for service attitude is from 0 to 1, with a greater value indicating a higher service attitude.
**Moderating variables**		
	Hreputation	When patients post experiences, they also give a score on the hospital’s environment and attitude of guide service. The range of values for the hospital’s online reputation is from 0 to 10, with a greater value representing a higher level of satisfaction.
	Disease_severity	The severity of disease that patients get. We use one dummy variable to measure it. When the disease is high-risk, the variable is equal to 1.
**Control variables**		
	Dtitle_dummy1 and Dtitle_dummy2	Doctors’ medical skills as evaluated by the government, including Chief Doctor, Associate Chief Doctor, and Attending Doctor. Two dummy variables were used. (0, 0) represents Attending Doctor title or below.
	Hlevel_dummy	The variable indicating the comprehensive health care quality of doctor *i’*s affiliated hospital in terms of medical skills, equipment, human resources, etc. *Hlevel_dummy* presents AAA level and above hospitals. (0, 0) represents AA level hospital or below.

### Dependent Variable

The dependent variable in our model is patient propensity to post treatment experiences. The variable is the ratio of the increment of the treatment experience to the increment of outpatient care service demands over a certain time period. The dependent variable is defined as follows:



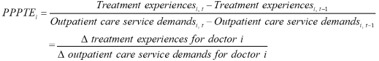



where *i* represents each doctor. The subscripts *t* and *t−1* denote 2 periods in time and PPPTE denotes patient propensity to post treatment experiences.

### Independent and Moderating Variables

The independent variable in our model is the doctor’s online reputation, which is divided into 2 dimensions: medical quality and service attitude. The WeDoctor website calculates the mean of medical quality and mean of service attitude for each doctor based on all the existing treatment experiences posted by patients. The range of values for both medical quality and service attitude is from 0 to 1, with a greater value indicating a higher satisfaction.

The moderating variables were the hospital’s online reputation and disease severity for patients treated in the hospital. The hospital’s online reputation reflects the integral medical quality and integral service attitude delivered by the hospital. The range of values for the hospital’s online reputation is from 0 to 10, with a greater value representing a higher level of satisfaction. We used mortality rates to distinguish the severity of different diseases. The Chinese Health Statistics Yearbook, published in 2019 [53], reports the latest health statistics, which lists mortality rates for different categories of diseases. We chose the first 3 fatal categories of diseases as severe diseases in our model. They are malignant tumor–related diseases (mortality rate: 163.18/100,000), heart diseases (mortality rate: 146.34/100,000), and cerebrovascular diseases (mortality rate: 128.88/100,000). For less severe diseases, we chose endocrine-related diseases (mortality rate: 21.15/100,000), digestive system diseases (mortality rate: 14.54/100,000), and nervous system diseases (mortality rate: 8.62/100,000). The difference in mortality rates between severe diseases and less severe diseases is large (nearly 10 times), which is helpful for understanding the impact of disease severity on patient behaviors. We collected all active doctors who treat these diseases, and finally, 5602 doctors who treat severe diseases and 1581 doctors who treat less severe diseases are included. A dummy variable was used to measure disease severity, with 1 representing severe diseases and 0 representing less severe diseases:



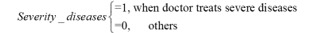



### Control Variables

We included both doctors’ titles and hospital levels in our model to control for their popularity offline. In China, each doctor has an offline title that represents their medical skills and level of experience, including Chief Doctor, Associate Chief Doctor, and Attending Doctor. These titles are evaluated and issued by government agencies. We use 2 dummy variables, Dtitle_dummy1 and Dtitle_dummy2, to measure doctors’ titles. Similarly, each hospital in China is assigned a rank, classified as class A, B, or C, with class A being the best quality of hospital. Hospital level, which is also evaluated and issued by government agencies, represents their medical quality and medical technical strength. As the number of class C hospitals in this online health community is very small, we combined it with class B and used 1 dummy variable, Hlevel_dummy, to measure hospital level. The detailed definitions of these dummy variables are as follows:



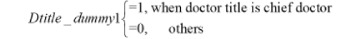





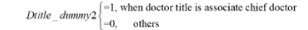





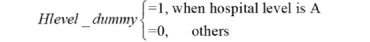



We use general linear model regression to obtain empirical results. Fractional logistic regression is most suitable for our dependent variable (% of patients posting treatment experiences). On the basis of all the hypotheses, the empirical models are as follows:

*Logit(PPPTE_i)=β_1,i_ Dtitle_dummy1+β_2,i_ Dtitle_dummy2+β_3,i_ Hlevel_dummy*

*+β_4,i_ Dmedical_quality_t-1_+β_5,i_ Dservice_attitude_t-1_+β_6,i_ Hreputation_t-1_*

+β_7,i_ Severity_diseases+β_8,i_ Dmedical_quality_t-1_*Hreputation_t-1_

+β_9,i_ Dservice_attitude_t-1_*Hreputation_t-1_

+β_10,i_ Dmedical_quality_t-1_*Severity_diseases

+β_11,i_ Dservice_attitude_t-1_*Severity_diseases+ε

where *i* represents each doctor. The subscripts *t* and *t−1* denote 2 periods in time. We use data collected at time=*t−1* for the independent variable and time=*t* for dependent variables.

## Results

### Descriptive Statistics and Correlations

We use the expectation-confirmation theory in our hypotheses to argue that patients have higher expectations when they choose doctors with high reputations. Patients are likely to feel disconfirmed between expectation and perceived quality of the service and express their feelings online.

Table 2 presents descriptive statistics and correlations for the key variables in our study. We can see that doctors’ reputation, hospitals’ reputation, and disease severity are correlated with patient propensity to post treatment experiences. Doctors’ reputation and the hospital’s reputation positively affect patient propensity to post treatment experiences; conversely, disease severity negatively impacts patient propensity to post treatment experiences. All variance inflation factor values, of all variables, are below 5, which indicates the negligible effect of multicollinearity.

**Table 2:** Description and correlation (N=7183).

Variables	Mean (SD)	Minimum	Maximum	Patient propensity to post treatment experiences	Dtitle_dummy1	Dtitle_dummy2	Hlevel_dummy	Dmedical_quality	Dservice_attitude	Hreputation
Patient propensity to post treatment experiences	0.067 (0.108)	0	0.944	—^a^	—	—	—	—	—	—
Dtitle_dummy1	0.348 (0.476)	0	1	0.229^b^	—	—	—	—	—	—
Dtitle_dummy2	0.443 (0.496)	0	1	−0.050^b^	−0.591^b^	—	—	—	—	—
Hlevel_dummy	0.088 (0.283)	0	1	−0.180^b^	−0.016^b^	0.037^b^	—	—	—	—
Dmedical_quality	0.338 (0.442)	0	1	0.790^b^	0.197^b^	−0.038^b^	−0.167^b^	—	—	—
Dservice_attitude	0.402 (0.471)	0	1	0.860^b^	0.208^b^	−0.044^b^	−0.175^b^	0.889^b^	—	—
Hreputation	5.740 (4.192)	0	10	0.477^b^	0.082^b^	−0.055^b^	−0.328^b^	0.447^b^	0.486^b^	—
Severity_diseases	0.780 (0.414)	0	1	−0.329^b^	0.080^b^	−0.022^b^	−0.587^b^	0.280^b^	0.309^b^	0.537^b^

^a^This table is symmetrical. The number in the lower left corner is same as the at top right corner.

^b^Correlation is significant at the .01 level (2-tailed), significant at .01

### Empirical Results

The analyses are deemed fit using Stata, a data analysis software. The empirical results are shown in Table 3. Model 1 contains all the control variables. Model 2 adds all the independent variables. Model 3 adds all the moderating variables, and model 4 adds all the interaction terms of the independent variables and moderating variables. We also tested the interactions between doctors’ reputation, hospital reputation, and disease severity in model 5. As none of the interaction terms are significant, model 5 is not further discussed.

Hypotheses 1a and 1b concern the impact of the doctor’s reputation on patient propensity to post treatment experiences. From model 4 in Table 3, we see that both medical quality and service quality have a positive impact on this dependent outcome. Patients are more likely to post reviews about their treatment experiences for those doctors who have a higher reputation. Moreover, the effect size of service attitude is nearly 4 times that of medical quality (β_service attitude_=.233; *P*<.001 and β_medical quality_=.052; *P*<.001), which indicates that service attitude plays a more important role in influencing patient propensity to post treatment experiences than medical quality; thus, both hypotheses 1a and 1b are supported.

As the results of model 4 show that a hospital’s reputation has no significant impact, we focused on its margin effect, with results demonstrating that a hospital’s reputation has a positive influence on patient propensity to post treatment experiences (β=.001; *P*<.001). Patients are more likely to post about their treatment experiences for those doctors who work in hospitals with a high reputation. Thus, hypothesis 2a is supported. Disease severity had a negative impact on patient propensity to post treatment experiences (β=.004; *P*=.009). People with more severe diseases are less likely to post reviews online. Thus, hypothesis 3a is supported.

Hypotheses 2b and 2c test the moderating effects of organizational reputation on the relationship between individual reputation and consumer behavior. From model 4 in Table 3, we observe that a hospital’s reputation positively moderates the relationship between medical quality and patient propensity to post treatment experiences (β=.002; *P*=.01) and negatively moderates the relationship between service attitude and patient propensity to post treatment experiences (β=.004; *P*=.01). The impact of medical quality on patient propensity to post treatment experiences is greater for doctors who work in hospitals with higher reputations, whereas the impact of service attitude on patient propensity to post treatment experiences is smaller for doctors who work in hospitals with higher reputations; thus, hypothesis 2b is supported, whereas hypothesis 2c is not.

Hypotheses 3b and 3c examine the moderating effects of consumer characteristics (disease severity) on the relationship between individual reputation and consumer behavior. From model 4 in Table 3, we see that disease severity not only negatively moderates the relationship between medical quality (β=.036; *P*<.001) and patient propensity to post treatment experiences but also negatively moderates the relationship between service attitude (β=.044; *P*<.001) and patient propensity to post treatment experiences. The impact of both medical quality and service attitude on patient propensity to post treatment experiences is smaller for doctors who treat severe diseases; thus, both hypotheses 3b and 3c are supported.

To better interpret our results, we use the empirical results for the dependent variable, the increment of outpatient care service demands, and take its log value in the empirical model. The results are shown in Table 4. The impact of medical quality on patients’ choice was greater for patients with severe diseases than for those with less severe diseases (β=.319; *P*=.04). Severe diseases increase the demand for outpatient care services and positively moderate the relationship between doctors’ reputation and outpatient care service demands. On the contrary, for patient propensity to post treatment experiences, our results show that doctors who treat severe diseases are less likely to receive reviews about treatment experience (β=.325; *P*=.05). Moreover, the moderating effects of disease severity on doctors’ reputation are negative.

**Table 3:** Results for the patient propensity to post treatment experiences. General linear model regression was used to obtain results.

Variables	Model 1, coefficient (SD)	Model 2, coefficient (SD)	Model 3, coefficient (SD)	Model 4, coefficient (SD)	Model 5, coefficient (SD)
Constant	0.054^a^ (0.006)	0.012^b^ (0.003)	0.026^a^ (0.003)	0.012^a^ (0.004)	0.012^a^ (0.004)
Dtitle_dummy1	0.031^a^ (0.015)	−0.012^a^ (0.008)	−0.011^a^ (0.008)	−0.010^a^ (0.008)	−0.010^a^ (0.008)
Dtitle_dummy2	0.015^a^ (0.015)	−0.006^a^ (0.008)	−0.005^a^ (0.008)	−0.004^a^ (0.008)	−0.004^a^ (0.008)
Hlevel_dummy	−0.041^a^ (0.025)	0.004^c^ (0.014)	−0.012^c^ (0.015)	−0.013^a^ (0.015)	−0.013^a^ (0.015)
Dmedical_quality	N/A^c^	0.013^a^ (0.015)	0.013^a^ (0.015)	0.076^a^ (0.022)	0.052^a^ (0.012)
Dservice_attitude	N/A	0.157^a^ (0.014)	0.158^a^ (0.014)	0.222^a^ (0.019)	0.233^a^ (0.019)
Hreputation	N/A	N/A	0.001^a^ (0.001)	0.001 (0.001)	0.001 (0.001)
Severity_diseases	N/A	N/A	−0.024^a^ (0.011)	−0.004^d^ (0.001)	−0.004^d^ (0.001)
Dmedical_quality×Hreputation	N/A	N/A	N/A	0.002^d^ (0.001)	0.008^a^ (0.002)
Dservice_attitude×Hreputation	N/A	N/A	N/A	−0.004^d^ (0.002)	−0.007^a^ (0.002)
Dmedical_quality×Severity_diseases	N/A	N/A	N/A	−0.036^a^ (0.014)	−0.039^a^ (0.014)
Dservice_attitude×Severity_diseases	N/A	N/A	N/A	−0.044^a^ (0.012)	−0.063^a^ (0.012)
Dmedical_quality×Hreputation×Severity_diseases	N/A	N/A	N/A	N/A	−0.009 (0.014)
Dservice_attitude×Hreputation×Severity_diseases	N/A	N/A	N/A	N/A	0.004 (0.012)
Log likelihood	−4790.53	−4531.46	−4233.40	−3610.23	−3577.21
Pseudo-*R*^2^	0.014	0.015	0.018	0.020	0.021

^a^Significant at .001.

^b^Significant at .05.

^c^N/A: not applicable.

^d^Significant at .01.

**Table 4:** Results for the increment of outpatient care service demands. Ordinary least squares regression was used to obtain results.

Variables	Model 1
Constant	−0.188^a^ (0.018)
Dtitle_dummy1	0.399^a^ (0.015)
Dtitle_dummy2	0.143^a^ (0.014)
Hlevel_dummy	0.039^b^ (0.023)
Dmedical_quality	0.341^c^ (0.110)
Dservice_attitude	1.859^a^ (0.098)
Hreputation	0.018^a^ (0.002)
Severity_diseases	0.029 (0.019)
Dmedical_quality×Hreputation	0.051^c^ (0.020)
Dservice_attitude×Hreputation	0.050^c^ (0.018)
Dmedical_quality×Severity_diseases	0.319^b^ (0.148)
Dservice_attitude×Severity_diseases	−0.325^b^ (0.131)
Adjusted *R*^2^	0.785

^a^Significant at .001.

^b^Significant at .05.

^c^Significant at .01.

### Robustness Check

In our study, it was found that many doctors did not receive any reviews from patients, which may have caused bias in our findings. A small increment in treatment experiences will not change the doctor’s reputation too much [17]. To check the robustness of our findings, we only included doctors whose increments of treatment experiences were equal to or greater than 1, 5, and 10. The results of the sensitivity analyses are shown in Table 5. As the results are almost identical to those shown in Table 3, our findings are deemed quite robust.

**Table 5:** Robustness check results. General linear model regression was used to obtain results.

Variables	The increment of treatment experiences ≥1; n=4461	The increment of treatment experiences ≥5; n=2462	The increment of treatment experiences ≥10; n=1651
Constant	0.351^a^ (0.014)	−0.055 (0.085)	−0.319 (0.275)
Dtitle_dummy1	−0.029^a^ (0.002)	−0.037^a^ (0.003)	−0.043^a^ (0.003)
Dtitle_dummy2	−0.017^a^ (0.002)	−0.028^a^ (0.003)	−0.033^a^ (0.003)
Hlevel_dummy	−0.106^a^ (0.006)	−0.178^a^ (0.010)	−0.179^a^ (0.012)
Dmedical_quality	0.014^b^ (0.010)	0.108^a^ (0.066)	0.489^a^ (0.154)
Dservice_attitude	0.028^b^ (0.016)	0.299^a^ (0.155)	0.718^c^ (0.288)
Hreputation	−0.012^a^ (0.001)	−0.018 (0.008)	0.112 (0.027)
Severity_diseases	−0.121^a^ (0.016)	−0.252^a^ (0.061)	−0.170^c^ (0.030)
Dmedical_quality×Hreputation	0.007^c^ (0.002)	0.001^c^ (0.000)	0.055^b^ (0.023)
Dservice_attitude×Hreputation	−0.006^b^ (0.002)	−0.017^c^ (0.002)	−0.032^c^ (0.018)
Dmedical_quality×Severity_diseases	−0.021^c^ (0.014)	−0.147^c^ (0.080)	−0.234^a^ (0.063)
Dservice_attitude×Severity_diseases	−0.004^a^ (0.001)	−0.414^a^ (0.101)	−0.206^c^ (0.103)
Log likelihood	−4021.50	−5112.12	−5825.11

^a^Significant at .001.

^b^Significant at .01.

^c^Significant at .05.

## Discussion

### Principal Findings

This study provides valuable insights into the impact factors of sharing patient reviews in online health care communities. We study the impact of individual reputation, organizational reputation, and consumer characteristics on patient propensity to post treatment experiences and the moderating effects of organizational reputation and patient characteristics. From our results, most of the hypotheses are supported.

Our findings suggest that both medical quality and service attitude positively impact patient propensity to post treatment experiences, which is consistent with the expectation-confirmation theory [31] and existing literature [17,34]. When patients choose doctors with a high reputation, they hold higher expectations and are more likely to be disappointed after receiving treatment services. Similarly, when patients choose doctors who work in hospitals with high reputation, they again have high expectations, decreasing the possibility of confirmation between expectation and perceived performance.

**Figure 4:**
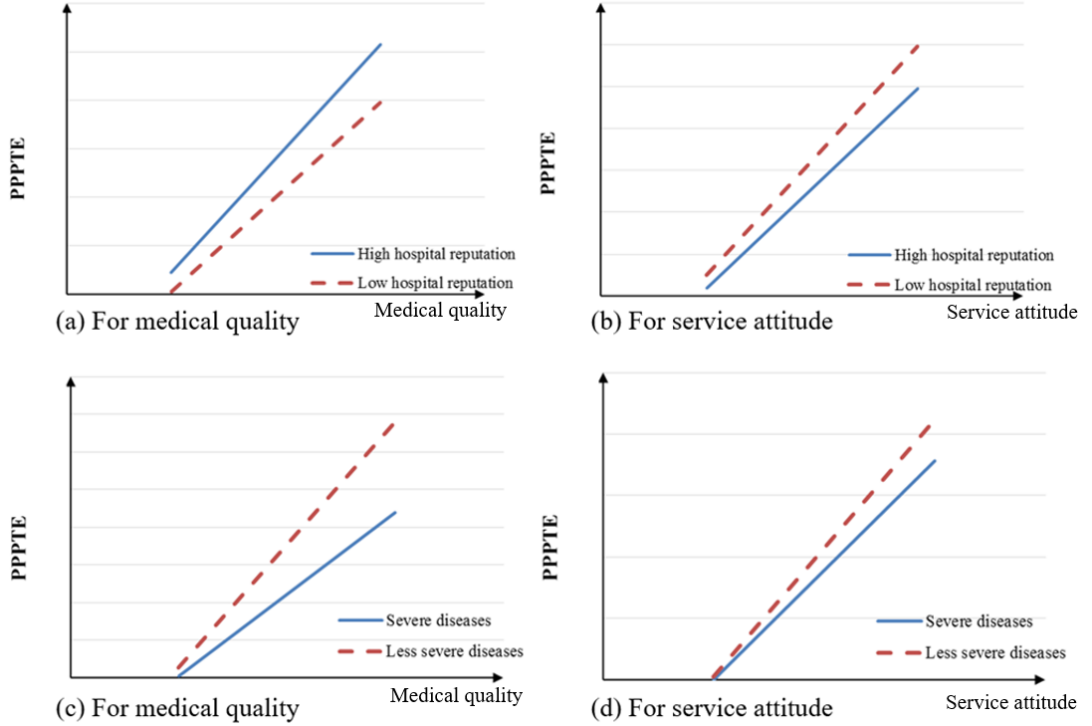
The moderating effects of hospitals’ reputation and disease severity. PPPTE: patient propensity to post treatment experiences.

Our results provide further evidence for the theory of psychological choice [18], as we explore the moderating effects of environmental factors in health care. The interaction effects are illustrated in Figure 4. Hospitals with high reputations can minimize patients’ perceived risks and increase trust in doctors’ reputations, which is confirmed in our results; a hospital’s reputation positively moderates the relationship between a doctor’s medical quality and patient propensity to post treatment experiences. However, a hospital’s reputation negatively moderates the relationship between doctors’ service attitude and patient propensity to post treatment experiences. In China, patients prefer to choose hospitals with high reputation and make appointments with doctors who also have a high reputation. As a result, hospitals with a high reputation for both their facilities and doctors are under tremendous pressure and constantly overloaded; for this reason, patients have difficulty in making appointments with these hospitals and doctors. Due to excessive number of patients, such hospitals often have low service attitudes. Therefore, patients often have low expectations about service attitude when choosing doctors who work in hospitals with high reputation.

### Strengths and Limitations

In this study, we examine the theory of psychological choice [18] by researching the moderating effects of consumer characteristics. The results are consistent with our a priori hypothesis, and the possible explanations are as follows. First, medical services employing specialized knowledge and technology are difficult for patients to evaluate, especially for severe diseases. Consequently, following the decrease in certainty of disconfirmation between expectation and perceived quality, patient propensity to post treatment experiences decreases. Second, patients with severe diseases are more concerned about their recovery and health, instead of posting reviews. Third, for the protection of privacy and information security, patients with severe diseases may not post reviews about their treatment experiences.

Our study makes several contributions to the literature. First, this is one of the earliest in-depth studies to analyze the role of reputation in patient propensity to post treatment experiences. Prior studies have focused on the relationship between reputation and sellers’ sales in both product fields [6] and health care [19]. In our study, we explore how reputation influences patient propensity to post treatment experiences, which is an important predictor of a seller’s future performance [6]. Our study broadens the knowledge base on how patients think about doctors and enriches the literature on the reputation and motivation of online reviews in health care.

Second, this study contributes to the existing literature on reputation by researching the role of individual reputation, organizational reputation, and interaction effects. Prior studies have only considered reputation at one level, either individual [54] or organizational [43]. In health care, Wu and Lu [17] researched the impact of individual reputation on patients’ propensity to post reviews, but they failed to consider organizational reputation. Both individual and organizational reputations work effectively to determine consumer behavior, especially for medical services with high information asymmetry [55]. In the health care field, doctors are affiliated with hospitals. Patients often place great importance on a hospital’s reputation (environment of signal delivery), which must be considered in health care. Our study helps understand the role of doctors’ (individual) reputation, hospital (organizational) reputation, and their interaction effects on patient propensity to post treatment experiences.

Third, we enrich the existing literature on the impacts of consumer characteristics on consumer behavior. Consumer characteristics have been recognized by researchers as one of the most influential factors for different consumer behaviors [19,27]. Among these patient characteristics, disease severity is extremely important. However, there is not much current investigation into the impact of patient characteristics on their behavior, which is measured by their propensity to post reviews about treatment experiences. Our research provides an empirical analysis of the theory of psychological choice by examining the moderating effects of disease severity on whether patients post their experiences.

This study also has significant practical implications. First, our results show that when patients decide whether to post treatment experience reviews, service attitude works more effectively than medical quality. Our findings also suggest that doctors need to pay more attention to their service attitude than ever before. When people have diseases, they become vulnerable and seek emotional support from doctors. Moreover, contradictions and disputes between doctors and patients have intensified, which has reached an unprecedented level in recent years, requiring doctors to improve their service attitudes. Second, not only do we find that doctors’ reputation has a positive impact on the number of reviews posted but also the hospital’s reputation; thus, to encourage more patients to post reviews online, doctors must take the impact of the hospital’s reputation into consideration. For example, doctors can move to other hospitals with higher reputation. Third, disease severity mitigates the relationship between doctor reputation and patient propensity to post treatment experiences. Compared with doctors who treat severe diseases, doctors who treat less severe diseases should pay closer attention to their online reputation. As their online reputation increases, doctors who treat less severe diseases receive a greater number of patient reviews than those who treat severe diseases.

Our study has several limitations. First, we include one online service, the WeDoctor website. Although improving the internal validity, this choice may reduce the generalizability of our findings. Other contexts should be examined in future studies. Second, we did not collect patient-level data; because of this, we could not measure demographic characteristics and specific disease severity for each patient. Future research can improve our findings by collecting data at the patient level. Third, we did not analyze the content of treatment experience reviews. These new treatment experiences may reflect different feelings and play different roles and should be investigated in future studies. Last but not least, future studies should adopt a longitudinal approach to improve our findings by addressing potential endogeneity issues and dynamic effects.
